# Trimethylamine N-oxide (TMAO) acutely alters ionic currents but does not increase cardiac cell death

**DOI:** 10.3389/fphys.2025.1505813

**Published:** 2025-02-13

**Authors:** Simona Esposito, Lauren R. McGuinness, Parveen Sharma, Amy E. Chadwick, Richard D. Rainbow

**Affiliations:** ^1^ Department of Cardiovascular Sciences, Glenfield General Hospital, University of Leicester, Leicester, United Kingdom; ^2^ Department of Cardiovascular & Metabolic Medicine and Liverpool Centre for Cardiovascular Sciences, Institute of Life Course and Medical Sciences, University of Liverpool, Liverpool, Merseyside, United Kingdom; ^3^ Department of Pharmacology and Therapeutics, Molecular and Integrative Biology, Institute of Systems, Liverpool, Merseyside, United Kingdom

**Keywords:** trimethylamine N-oxide, calcium channels, cardiac ATP-sensitive potassium channels, Kir6.1, biomarker

## Abstract

**Background:**

Trimethylamine N-oxide (TMAO) is a product of the action of gut microbiota on choline and other choline-containing compounds ingested in the diet. The presence of TMAO at high concentrations has been reported in the blood of patients with cardiovascular disease, suggesting the role for TMAO as either a marker or causative agent of the disease. These investigations examined whether TMAO had an effect on cardiomyocyte contractile function, calcium homoeostasis, and survival from metabolic insult.

**Results:**

TMAO had no effect on metabolic function or the ability of cells to survive a metabolic insult; however, it did cause transient changes to contractile function. These changes included an increase in calcium current and an increase in Kir6.1 channel activity in the cell, causing a shortening of the action potential duration to 90% repolarised but lengthening the action potential to 30% repolarised. These effects occurred within minutes of TMAO application; however, they were not observed following 24 h culture. These data suggest that TMAO does modulate contractile function, albeit only in the short-term, but has no effect on metabolic behaviour or the ability to withstand a metabolic challenge.

**Conclusion:**

These data suggest that high TMAO concentrations in the blood of patients may be a marker of potential cardiovascular disease rather than playing a causative role.

## Introduction

The gut microbiome plays an important role in health and disease. Patients with cardiovascular disease often display a change in their gut microbiota, where there is an increased production of pro-inflammatory molecules (Nesci et al., 2023; Rahman et al., 2022; Masenga et al., 2022; Hassan et al., 2023). Furthermore, there have been links between the severity of myocardial infarction and intestinal flora, for example, the concentrations of leptin are reduced in patients on broad-spectrum antibiotics, which can lead to a reduced infarct size in acute coronary syndromes (Lam et al., 2016; Lam et al., 2012).

Trimethylamine N-oxide (TMAO) is produced from trimethylamine (TMA) and arises when bacteria residing in the human gut flora metabolise choline and other choline-containing compounds, such as betaine and L-carnitine, ingested in the diet. The action of gut microbiota is required for the conversion of these nutrients into TMA (al-Waiz et al., 1992; Romano et al., 2015), evidenced by a reduction in TMA levels in patients on broad-spectrum antibiotics (al-Waiz et al., 1992). Around 95% of TMA is oxidized into TMAO, and excessive oxidisation to TMAO can give rise to a distinct “fish odour” in patients with high TMAO concentrations (Zhang et al., 1995). TMA is oxidised to TMAO in the liver by hepatic flavin monooxygenases. High levels of TMAO are linked to liver disease, such as steatosis and metabolic-associated fatty liver disease (Florea et al., 2024). Furthermore, elevated TMAO levels can inhibit cholesterol and bile acid metabolism (Florea et al., 2024). Plasma TMAO levels are typically 10 to 20 times higher than TMA (Seldin et al., 2016). These levels exhibit significant inter- and intra-individual variations influenced by factors including diet, gut microbiome, liver flavin monooxygenase activity, kidney function, and age (Velasquez et al., 2016). In healthy individuals, plasma TMAO levels range from 1 to 20 μmol/L in men and 1–17 μmol/L in women (Gessner et al., 2020). The primary sources of L-carnitine and other TMAO precursors are animal products. Meat, poultry, eggs, and milk contain the highest levels of L-carnitine, whereas plants contain little to none (Flanagan et al., 2010).

Plasma and serum circulating levels of TMAO have been associated with different cardiovascular diseases and are also linked to the prediction of incident risk for adverse cardiovascular events, such as myocardial infarction, stroke, and death, when used in conjunction with other traditional cardiac risk factors and evaluation of renal function (Tang et al., 2013; Wang et al., 2011). Alongside TMAO, plasma levels of choline, betaine, and carnitine have also been used as predictors of adverse cardiac events and have been linked with an increased CVD risk, but their relevance and predictive value seem to be dependent on the serum levels of TMAO (Wang et al., 2014; Koeth et al., 2013). The link between elevated TMAO levels and incidence of adverse cardiac events has been shown in several studies; however, whether this is causative, or merely a marker of poor health, remains unclear (Tang et al., 2013; Wang et al., 2011; Tang and Hazen, 2014; Tang et al., 2015; Senthong et al., 2016a; Canyelles et al., 2023; Lee et al., 2021; Andraos et al., 2021). Elevated plasma concentrations of TMAO have also been linked with a long-term mortality risk in patients suffering from heart failure, advanced left-ventricular diastolic dysfunction, and atherosclerotic development in patients suffering from atherosclerotic coronary artery disease (Tang et al., 2013; Wang et al., 2014; Tang et al., 2015; Senthong et al., 2016a; Lee et al., 2021; Senthong et al., 2016b). Finally, high circulating TMAO levels were also associated with the presence of vulnerable coronary plaque, plaque rupture, and long-term risks of incident cardiovascular events in patients with acute coronary syndrome (Tang et al., 2017; Fu et al., 2016; Li et al., 2017). Overall, these findings indicate that increased levels and accumulation of TMAO have detrimental effects on CVD.

The cardiac ATP-sensitive potassium (K_ATP_) channel has been associated with both myocardial protection and toxicity. Under normal physiological conditions, these channels are inactive due to ATP inhibition (Inagaki et al., 1995; Inagaki et al., 1996). During severe metabolic stress, such as ischaemia (Noma, 1983), ventricular Kir6.2/SUR2A channels open, leading to shortened action potential duration, reduced Ca^2+^ entry, and decreased contractile activity as essential energy-saving protective mechanisms (Nichols, 2016; Cole et al., 1991; Suzuki et al., 2002). Recently, a second cardiac K_ATP_ channel family member was identified at the ventricular cell surface, with a Kir6.1 pore (Brennan et al., 2024). Unlike the Kir6.2/SUR2A channels, this channel is constitutively active under normal conditions and plays a crucial role in modulating the action potential duration and resting membrane potential (Brennan et al., 2024). Blocking this channel results in a prolonged action potential, while potentiation leads to a shortened action potential. Both of these changes show potential to be proarrhythmic. Given the metabolic dysregulation associated with TMAO and the dependence of the K_ATP_ channel on metabolic and kinase pathway regulation, it is plausible that TMAO could be involved in cardiotoxicity through a K_ATP_ channel-dependent mechanism.

This study employed *in vitro* and *ex vivo* methods to explore whether TMAO serves as a marker or plays a causative role in cardiac damage. This was assessed by investigating the functional characteristics of rat isolated ventricular myocytes, or whole rat hearts, exposed to increasing concentrations of TMAO. It was hypothesised that if TMAO is cardiotoxic contributing to worse outcomes for patients, its presence would increase cell death and limit contractile recovery in simulated ischaemia assays. Furthermore, an increase in infarct size would be anticipated in whole heart coronary ligation experiments. To assess the effects of TMAO on cardiac contractile properties, the amplitude and duration of contractions were investigated using video-edge detection experiments. Patch clamp recording was used to study action potential properties and the behaviour of the underlying currents. Additionally, calcium signalling involved in the cardiac contractile cycle was measured.

## Materials and methods

### Ethical approval

All methods were carried out in accordance with relevant guidelines and regulations. Adult male Wistar rats (200–300 g, 10–14 weeks old, n = 96 in total, purchased from Charles River Laboratories, United Kingdom, and used at the Universities of Leicester or Liverpool) were killed by concussion and cervical dislocation. Animals were maintained in 1,800-cm^2^ Tecniplast cages, using BED01/8 dates and corn cob for bedding, housed in individually ventilated caging (specific pathogen-free). The care and death of the animals conformed to the requirements of the United Kingdom Animals (Scientific Procedures) Act 1986 (SI 2012/3039). All procedures were approved by the Universities of Leicester (AWERB_2018_44) and Liverpool (AWC0152-AWERB) animal welfare ethical review board. The ARRIVE guidelines for reporting experiments involving animals have been followed in this study.

### Sex as an experimental variable

The effect of sex and hormones on cardiovascular function is an area of ongoing research (Blenck et al., 2016; Chicco et al., 2007). To minimise any confounding effects of the sex and hormones of the animals, this study has solely used male animals as female animals demonstrate inherent cardioprotection. This limitation is mentioned in the Discussion section.

### Solutions

All solutions used in the perfusion of isolated cells or whole hearts were based on modified Tyrode’s solution, as used in our previous publications (Brennan et al., 2024; Brennan et al., 2019; Brennan et al., 2015). The basic solution contained (in mM) 135 NaCl, 5 KCl, 0.33 NaH_2_PO_4_, 5 Na pyruvate, 5 glucose, 15 mannitol, 10 HEPES, 1 MgCl_2_, and 2 CaCl_2_ (pH 7.4) and is referred to as normal Tyrode’s (NT) solution throughout. Nominally, Ca^2+^-free Tyrode’s solution was used in cardiomyocyte isolation and contained as mentioned above; however, it included 0.6 μM EGTA and had no added CaCl_2_. The pipette solution for whole-cell electrophysiological recording contained (in mM) 30 KOH, 5 EGTA, 110 KCl, 10 HEPES, 1 MgCl_2_, 0.61 CaCl_2_, 1 ATP, 0.1 ADP, and 0.1 GTP (pH 7.2). The pipette solution for cell-attached patch recording contained (in mM) 140 KCl, 10 HEPES, 1 CaCl_2_, and 0.5 MgCl_2_ (pH 7.4). All chemicals were purchased from Merck, Gillingham, United Kingdom. In all cell experiments, the perfusion temperature was maintained at 32°C ± 2°C using Heat Wave 30 (Dagan Corporation, United States) perfusion systems. Langendorff and Seahorse respirometry experiments were carried out at 37°C.

### Isolation of cardiomyocytes

Adult male Wistar rats (200–300 g) were killed by concussion and cervical dislocation. The protocol for the isolation of cardiomyocytes was as described previously (Brennan et al., 2024). In brief, the heart was rapidly excised and submerged in ice-cold nominal Ca^2+^-free NT solution to arrest contractions and prevent clot formation. The heart was then rapidly cannulated via the aorta on Langendorff apparatus, and warmed (37°C) Ca^2+^-free NT was retrogradely perfused via the aorta to clear residual blood. Following 6 min of Ca^2+^-free NT perfusion, the solution was exchanged for the Ca^2+^-free NT solution supplemented with 0.82 mg/mL collagenase (Type I), 0.5 mg/mL protease (type XIV 15% Ca^2+^), and 1.67 mg/mL BSA prepared from factor V albumin (all purchased from Merck, Gillingham, United Kingdom) for 4–8 min until rod-shaped cardiomyocytes were visible in the perfusate. The solution was then exchanged for the Ca^2+^-free NT solution for 2 min to wash out the residual enzyme solution, and the heart was cut down and washed with the NT solution (2 mM Ca^2+^). Cardiomyocytes were mechanically dispersed from the tissue in a shaking water bath with this method, yielding 70%–90% rod-shaped cardiomyocytes. Isolated cardiomyocytes were stored in NT at room temperature in 60-mm culture dishes on a rocking platform to prevent pelleting of the cells. Cells isolated with this method are viable for up to 18–24 h, following isolation with no detriment to contractility. Cells were typically used within 9 h for studies of the acute effects of TMAO. To isolate cardiomyocytes that had been protected by an ischaemic preconditioning protocol, whole hearts were perfused on the Langendorff cannula with a modified NT solution, containing no pyruvate. Following 5 min of perfusion with NT (no pyruvate), perfusion was halted for 5 min. Perfusion was restarted for further 5 min, and this cycle was repeated three times, as previously described (Brennan et al., 2015; Rodrigo and Samani, 2008). On the final reperfusion, the solution was replaced with the nominal Ca^2+^-free NT solution and enzymatic digestion followed, as described above.

### Culture of isolated cardiomyocytes

Freshly isolated cardiomyocytes were pelleted in the NT solution and resuspended in DMEM with glutamax and 5 mM glucose, supplemented with 2% Pen/strep and non-essential amino acids. A volume of 500 μL of cell suspension was added to each well of a 6-well plate, and 2 mL of media was added to the well. Cardiomyocytes were incubated at 37°C in 5% CO_2_ for 24 h in the absence (control) or presence of 100 μM TMAO. Stocks of TMAO for culture were prepared in full media and diluted into the cell culture well to the final concentration.

### Culture of AC16 cells

AC16 (human cardiomyocytes) cells were maintained and cultured in Dulbecco’s modified Eagle’s medium (DMEM) supplemented with 10% foetal bovine serum (FBS) and 4 mM glutamine and penicillin (100 IU)/streptomycin (100 mg/mL). The cells were kept at 37°C under a humidified 5% CO_2_ atmosphere. Under these conditions, the cells achieved 80% confluence. The cells were used up to passage 20. The cells were then seeded on to appropriate assay plates at appropriate seeding densities into T75 flasks or 96-well plates for the assays.

### Culture of Chinese hamster ovary (CHO) cells

Chinese hamster ovary (CHO) cells stably expressing hKir6.1 and hSUR2B subunits were purchased from B’SYS (Witterswil, Switzerland). CHO cells were maintained at 37°C in 5% CO_2_ in the F12 (HAM) medium supplemented with 10% foetal bovine serum, 1% penicillin/streptomycin solution, hygromycin 100 μg/mL, and puromycin 1 μg/mL for stable selection. Before use in patch clamp electrophysiology recordings, cells were treated with trypsin for 5 min, resuspended in PBS, and pelleted. The cells were then resuspended in the NT solution until use.

### Contractile function metabolic inhibition model

**FIGURE 1:**
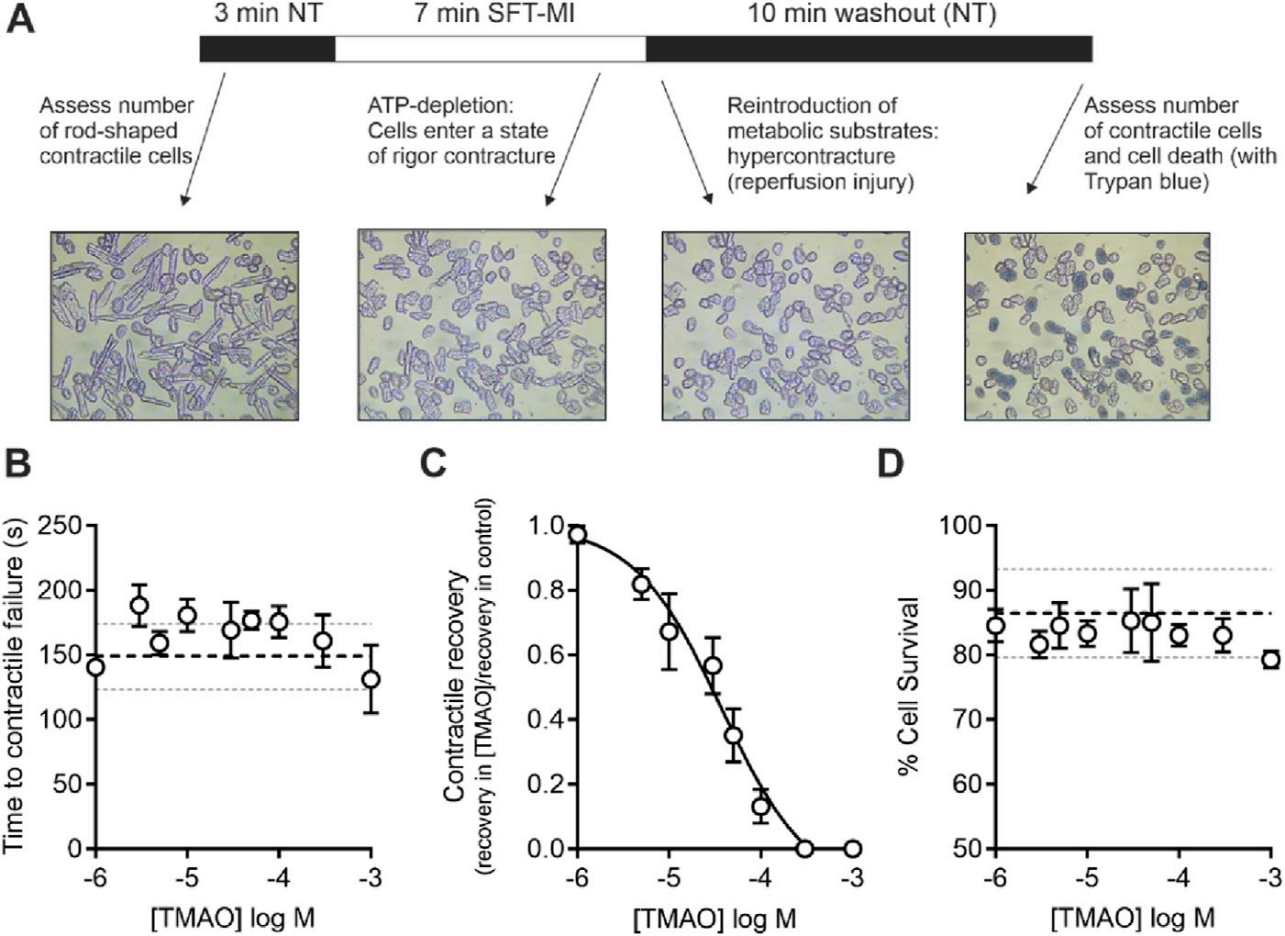
Increasing concentrations of TMAO inhibit contractile recovery but not cell survival following metabolic inhibition and washout. **(A)** Schematic representation of metabolic inhibition and washout protocol with key markers of the protocol identified. **(B)** Mean time to contractile failure with increasing concentrations of TMAO. The black dashed line indicates the mean time to contractile failure in control conditions, with the grey dotted lines indicating standard deviation (n = 18 animals (>5 animals per concentration)). **(C)** Mean contractile recovery data showing the reduction in contractile recovery in TMAO expressed as a fraction of the control recovery on that day (IC_50_ = 37 μM, n = 18 animals (>5 animals per concentration). **(D)** Mean percentage cell survival with increasing TMAO concentration. The black dashed line indicates the mean time to contractile failure in control conditions, with the grey dotted lines indicating the standard deviation (n = 18 animals (>5 animals per concentration)).

Cardiomyocytes were perfused at a rate of 5 mL/min maintained at a temperature of 32°C ± 2°C using a Dagan HW-30 perfusion chamber (United States) and were stimulated to contract using electric field stimulation (EFS) at 1 Hz via a Digitimer DS stimulator (Welwyn Garden City, United Kingdom). Contractile cells were defined as rod-shaped cells contracting synchronously with the EFS. Videos of the experiments were recorded using an iPod (Apple, United States) with a LabCam ultra microscope adaptor (United States) mounted on a Nikon inverted TMS microscope. Videos were transferred to a computer as mp4 files, and the contractile function data were analysed offline. For metabolic inhibition studies, the substrate-free Tyrode’s (SFT) solution was used, containing, in mM, 140 NaCl, 5 KCl, 0.33 NaH_2_PO_4_, 10 HEPES, 20 sucrose, 1 MgCl_2_, and 2 CaCl_2_ (pH 7.4). This SFT solution was supplemented with 1 mM iodoacetic acid and 2 mM sodium cyanide to make the metabolic inhibition solution (SFT-MI) (Brennan et al., 2019; Brennan et al., 2015) (Figure 1). In brief, cells were perfused for 3 min with the NT solution, 7 min with the SFT-MI solution, and washed out for a final 10 min with the NT solution. The time to contractile failure in the SFT-MI solution, the percentage of contractile recovery at the end of 10 min of washout, and the cell survival, as measured by trypan blue exclusion at the end of the 10 min of washout, were recorded. Only cardiomyocytes that were rod-shaped and contractile at the start of the recording were used in the analysis. It should also be noted that isolated cardiac cells in this model do not re-lengthen on washout as the cells are dissociated, so they do not have the stretch that would be experienced within the intact myocardium of blood refilling the ventricular chambers (see Figure 1A).

### Video-edge detection recording of contractile function in the absence of metabolic inhibition

To investigate the changes in the contractile function of isolated cardiomyocytes, videos were recorded using a Panasonic DVD recorder (Panasonic, Kadoma, Osaka, Japan) via a JC CCTV camera (JVC, Yokohama, Kanagawa, Japan). Videos were replayed through a video-edge detection system (VED-105, Crescent Electronics). The output was digitised using a MiniDigi 1B instrument (Molecular Devices) and recorded using AxoScope 10.7 software (Molecular Devices). Video-edge detection data were calibrated to a graticule and contraction, expressed as contraction in μm. As with the metabolic inhibition recordings, cells were perfused at a rate of 5 mL/min maintained at a temperature of 32°C ± 2°C using a Dagan HW-30 perfusion chamber (United States) and were stimulated to contract using electric field stimulation (EFS) at 1 Hz via a Digitimer DS stimulator (Welwyn Garden City, United Kingdom). Contractile cells were defined as rod-shaped cells contracting synchronously with the EFS.

### Seahorse XFe96 analyser

AC16 cells used in this study as a cardiomyocyte model for respirometry experiments have been widely used to assess cardiotoxicity in the literature (Reis-Mendes et al., 2024; Wang et al., 2023; Lippi et al., 2020). AC16 cells were seeded at 1.5 × 10^4^ cells/well in the Seahorse Bioscience 96-well plate and incubated for 24 h in a 37°C humidified incubator with 5% CO_2_.

In addition, 24 h before the experiment, 200 µL of Seahorse Bioscience Calibrant media (pH 7.4) was added to each well of the utility 96-well plate to hydrate the XFe96 sensor cartridge. The sensor cartridge was stored at 37°C without CO_2_ overnight. On the day of the experiment, DMEM culture media was removed from the AC16 96-well plate, and the cells were incubated at 37°C in 0% CO_2_ in unbuffered Seahorse XFe base media supplemented with 5 mM glucose, 1 mM Na pyruvate, and 2 mM L-glutamine. All TMAO concentrations were also made up in Seahorse XFe base media, as were the final optimised concentrations of the stress test compounds: oligomycin (1 μM), FCCP (0.25 μM), rotenone (1 μM), and antimycin A (1 μM). Normalised data obtained combining the XF stress test and BCA assay results were analysed using the Seahorse XF Mito Stress Test Generator from Agilent. Normalised OCR values were used to calculate non-mitochondrial respiration, basal respiration, proton leak, ATP-linked respiration, and spare respiratory capacity.

### Patch clamp recording

Whole-cell recordings were made from isolated cardiomyocytes using an Axopatch 200B amplifier (Axon instruments, Scientifica, Uckfield, United Kingdom), digitised using a Digidata 1440 instrument (Axon instruments, Scientifica, Uckfield, United Kingdom) and recorded and analysed using pCLAMP10.7 software (Axon instruments, Scientifica, Uckfield, United Kingdom). Cardiomyocytes were perfused with NT solution at 5 mL/min at 32°C ± 2°C. In the current-clamp mode, APs were stimulated at 1 Hz via the patch electrode with a 5-ms depolarizing trigger, set to 130% of that required to elicit an AP (approximately 500–900 pA). Action potential duration to 30, 50%, and 90% repolarized (APD_30_, APD_50_, and APD_90_) and membrane potential (Vm) were calculated within pCLAMP software offline (Brennan et al., 2019; Brennan et al., 2015). To measure different currents (I_Na_, I_Ca_, IKs, and IK_1_) from rat ventricular myocytes, voltage step protocols were used from a holding potential of −70 mV. To investigate the inwardly rectifying currents, inward Ca^2+^ currents, and outward delayed rectifier K^+^ currents, a voltage-step protocol was used that activated IK_1_ currents between −100 and −55 mV in 5 mV increments, a step to −50 mV to inactivate I_Na_, followed by a series of voltage steps between −40 and +60 mV, in 10-mV increments, to activate the Ca^2+^ and delayed rectifier K^+^ currents. To measure changes in calcium currents over time, a repeating voltage step from −50 mV, to inactivate I_Na_ currents, to 0 mV every 5 s was used. The design of these two step protocols is shown in Figures 6, 7, with example currents.

To record whole-cell Kir6.1/SUR2B currents, CHO cells were held at 0 mV for the duration of the recording, and the outward K^+^ currents were measured. These cells, stably expressing Kir6.1/SUR2B, were used for these experiments to allow the measurement of the response of Kir6.1 in an overexpression system, given the difficulties in isolating the Kir6.1 whole cell current in cardiomyocytes. The Kir6.1 current can be isolated in cardiomyocytes at the single-channel using cell-attached patch recording. For these cell-attached patch recordings, the electrode was held at +40 mV for the duration of the recording. Under these conditions, the equilibrium potential for potassium is close to 0 mV, and the voltage across the membrane patch is approximately −110 mV (the sum of the electrode potential and resting potential of the cardiomyocytes, assumed to be ∼ −70 mV) so that single-K_ATP_ channel openings lead to inward currents (Brennan et al., 2019; Brennan et al., 2015).

To investigate channel activity, the channel open probability (P_O_) was calculated; however, given that more than one channel was observed in each patch, the number of levels and duration of activity at each level were measured:

$$T_{O}=\sum_{L=1}^{N}t_{oL}.$$

T_O_ represents the duration that the channel was open, where L represents different open levels, t_oL_ is the duration of time in each level, and N is the number of ion channels in the recording. Given that the true number of channels cannot be measured because of the stochastic behaviour of ion channels, the number of channels is taken as the maximum number of levels open during the total (T) duration of the recording. The open probability was, therefore, presented as NP_o_:

$${NP}_{O}=\frac{T_{O}}{T}.$$

The sampling rate for cell-attached recordings was 50 kHz (Brennan et al., 2019; Brennan et al., 2015). Given the variability of the NP_0_, the geometric mean of the day was calculated for each data point presented in the cell-attached configuration. Data are, therefore, reported as n = animals. In addition, 60 s of data were used to measure NPo for each cell.

The Kir6.2/SUR2A complex is the canonical cardiac K_ATP_ channel isoform. This channel is metabolically sensitive, sensing a depletion in ATP to pass a hyperpolarising current. To assess whether TMAO affected the activation of this channel, the time to channel opening in metabolic inhibition was measured. Time to opening was defined as the first burst of Kir6.2/SURA channel opening that was greater than 100 ms in duration. The Kir6.2/SUR2A complex has a conductance of 70–80 pS and is seen as ∼10.5 pA current in the conditions of the cell-attached recording. This is variable as the membrane potential can change in the presence of metabolic inhibition, and when the Kir6.2/SUR2A channel opens, there is significant hyperpolarisation of the membrane potential.

### Fluo-4 measurements of intracellular calcium transients

Isolated cardiomyocytes were incubated with 5 µM Fluo-4AM indicator at room temperature for 30 min. Cells were allowed to adhere to a glass coverslip mounted in a Dagan HW-30 heated perfusion chamber (Dagan Corp, United States) for 10 min before experimentation. Solutions were perfused at a rate of 5 mL/min and at 32°C ± 2°C. Cardiomyocytes were stimulated to contract using EFS at 1 Hz. Fluo-4 fluorescence was measured using 488-nm excitation light from a PTI-monochromator (PTI, Birmingham, NJ, United States) with emissions collected above 520 nm using an Andor Zyla 4.5 Camera (Oxford Instruments, United Kingdom) with images recorded using WinFluor software (John Dempster, University of Strathclyde). To record the Ca^2+^ transients, images were acquired at a rate of 50 frames per second, and 2 × 2 pixel binning was used to improve the signal-to-noise ratio, given the short exposure time (20 ms).

### Whole-heart *ex vivo* left anterior descending coronary artery ligation on a Langendorff system

To measure the infarct size in a whole-heart *ex vivo* model, the left anterior descending (LAD) coronary artery was ligated for 40 min, following 1 h of equilibration on a Langendorff perfusion system, perfused at 8 mL/min at a temperature of 37°C and bubbled with 100% O_2_. The ligature around the LAD was removed, following the 40 min of ischaemia, and the heart was reperfused for 3 h. Following relegation, 1% Evans Blue dye was perfused through the heart to identify areas unaffected by the ligation. The heart was then cut down, frozen for 2 h, and cut into eight slices using a scalpel blade and stained in a Na_2_HPO_4_/NaH_2_PO_4_ solution (∼2:1 ratio of 0.1 M stock solutions until pH 7.4) with 10 mg/mL 2,3,5-triphenyltetrazolium chloride (TTC) (Sigma-Aldrich) to identify the area at risk (AAR) and infarcted area (IA, white in appearance), respectively. The heart slices were weighed, scanned on both sides, and ImageJ was used to assess the percentage area at risk and infarct. Data were analysed by two researchers, including the experimenter. The additional researcher was blinded to the condition, and the mean of the analysis used for each heart in this study, as previously described (Brennan et al., 2019; Brennan et al., 2022).

### Analysis and statistics

All data were analysed using Excel 365 (Microsoft) and Prism 10 (GraphPad). Statistical analysis was carried out in Prism, with the statistical test used outlined in the figure legends. All data are reported as n = animal (cells) or in the case of CHO and AC16 cells as n = passage of cells. All statistical analysis is carried out by animal/passage. Data in bar charts are shown as mean, with all data points displayed, and standard deviation for non-paired data, and as means with data points connected by dashed lines for paired or repeated-measure analysis.

## Results

### Contractile recovery following metabolic inhibition is reduced but cell survival is unaffected by TMAO

**FIGURE 2:**
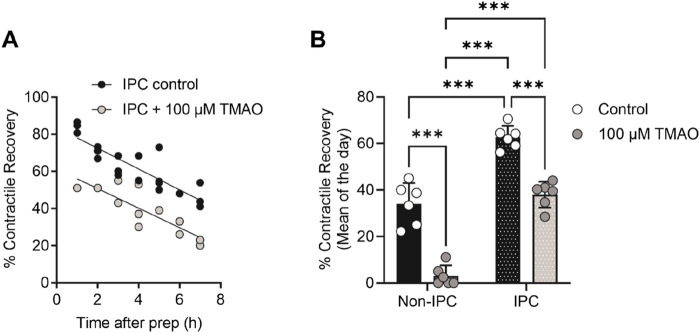
TMAO of 100 μM reduces the contractile recovery in cardiomyocytes protected, following an ischaemic preconditioning protocol. **(A)** Time course showing the percentage contractile recovery in individual experiments, following metabolic inhibition and washout in IPC cardiomyocytes in the absence and presence of 100 μM TMAO. Cardioprotection imparted by IPC wanes over time, so each data point is plotted as a duration after completion of the cell isolation. **(B)** Mean contractile recovery by day in non-IPC and IPC cardiomyocytes in the absence and presence of TMAO (***P < 0.0001, Two-way ANOVA with Tukey’s post-test, n = 6 animals in each group (16 and 11, 22 and 14 experiments, non-IPC and IPC in the absence and presence of TMAO, respectively).

Given the association of high TMAO concentrations in the blood and the incidence of cardiac disturbance in patients, it was hypothesised that increasing concentrations of TMAO would worsen the outcome for cardiac cells in a widely used metabolic inhibition and simulated reperfusion (MI/R) model (Figure 1A) (Brennan et al., 2019; Brennan et al., 2015). The timing of contractile failure has been shown to coincide with the opening of the canonical cardiac K_ATP_ channel, Kir6.2/SUR2A, as an indicator of severe ATP depletion (Brennan et al., 2019; Brennan et al., 2015). The timing of this failure and corresponding ATP depletion can demonstrate cardioprotection, with delayed contractile failure, or cardiotoxicity, with early contractile failure (Brennan et al., 2019; Brennan et al., 2015). In these experiments, the time to contractile failure was unchanged with increasing TMAO concentration (Figure 1B). The SFT-MI solution was washed out for 10 min with the NT solution, and contractile recovery (Figure 1C) and cell survival (Figure 1D) were assessed using Trypan Blue exclusion as an indicator. With increasing concentrations of TMAO in the perfusate, there was no significant change in the cell survival (Figure 1D); however, there was a TMAO concentration-dependent reduction in the contractile recovery when normalised to the control for the day (Figure 1C). The failure of contractile recovery was near complete at 100 μM TMAO (Figure 1C), and so this was investigated in cardiomyocytes that were cardioprotected, following an ischaemic preconditioning (IPC) protocol of the whole heart before cell isolation (Brennan et al., 2019; Brennan et al., 2015; Rodrigo and Samani, 2008). The window of protection afforded by IPC typically wanes within 8 h, following isolation (Figure 2A). In these cells, there was a significant reduction in contractile recovery in the presence of 100 μM TMAO (Figures 2A,B), but there was still some contractile recovery in the IPC cardiomyocytes in contrast to the unprotected cells where no contractile recovery was seen (Figure 2B).

### Infarct size in a whole-heart coronary ligation model was unaffected by 100 μM TMAO

**FIGURE 3:**
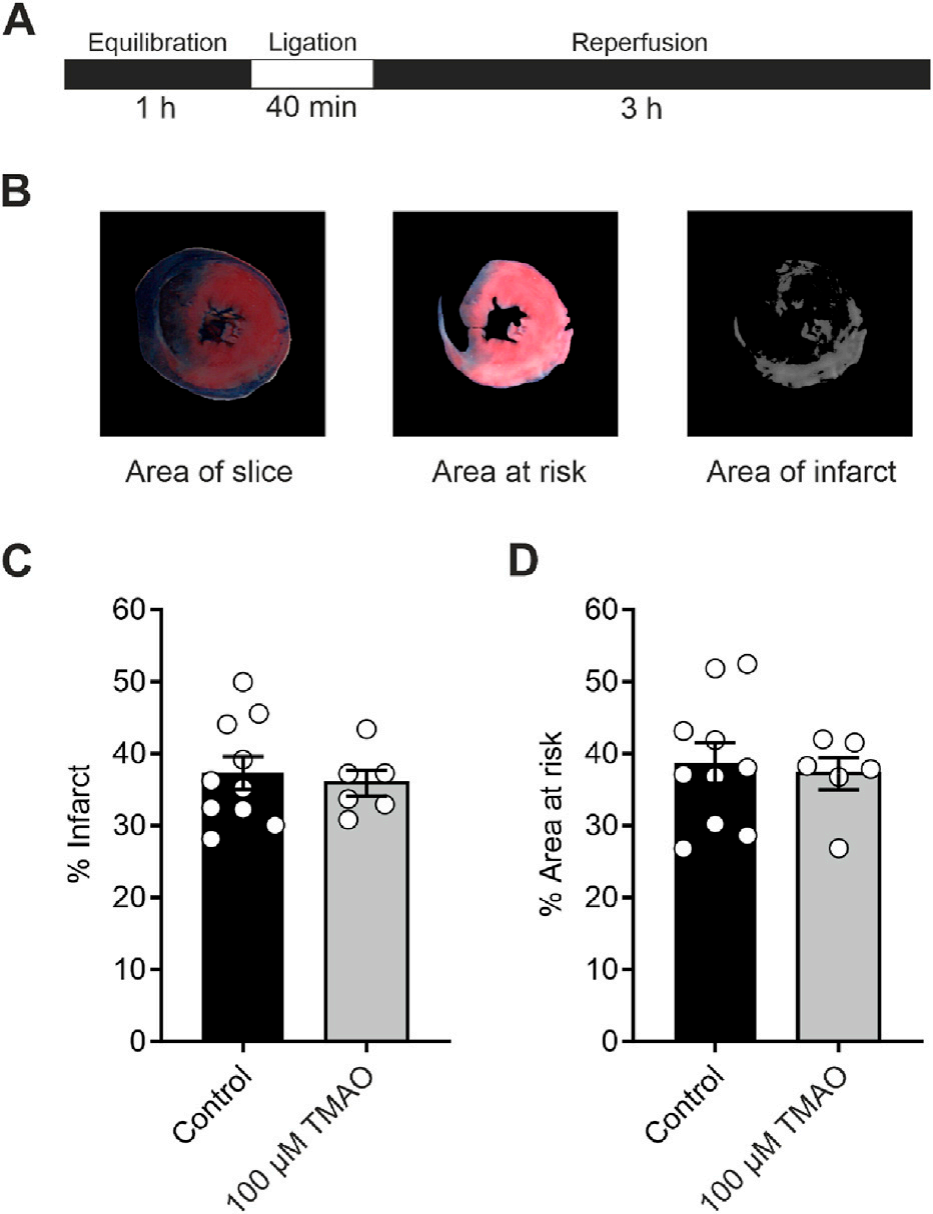
Infarct size in a whole-heart Langendorff coronary ligation model showed no significant difference in the presence of 100 μM TMAO. **(A)** Schematic representation showing the coronary ligation protocol used in this study. **(B)** Example image showing the ImageJ analysis used for the assessment of infarct size. **(C)** Mean percentage of infarct for each condition (each dot represents a single heart) (P = 0.67, unpaired t-test, n = 10 and 6 control and TMAO-treated hearts, respectively). **(D)** Mean percentage area at risk of the hearts used in **(C)** showing no significant difference (P = 0.72, unpaired t-test, n = 10 and 6 hearts in control and TMAO-treated, respectively).

To investigate whether TMAO could influence the damage imparted to a whole heart, a coronary ligation model was used (Figure 3A). The mean infarct size was no different in the control or the TMAO-treated groups (Figures 3B,C), while the mean area at risk between the two groups was also no different, demonstrating the consistency of the experiment (Figure 3D). These data confirm the findings in Figure 1 where there was no reduction in cell survival in the presence of TMAO.

### The metabolic profile of cardiomyocytes was unaffected by increasing TMAO concentrations

**FIGURE 4:**
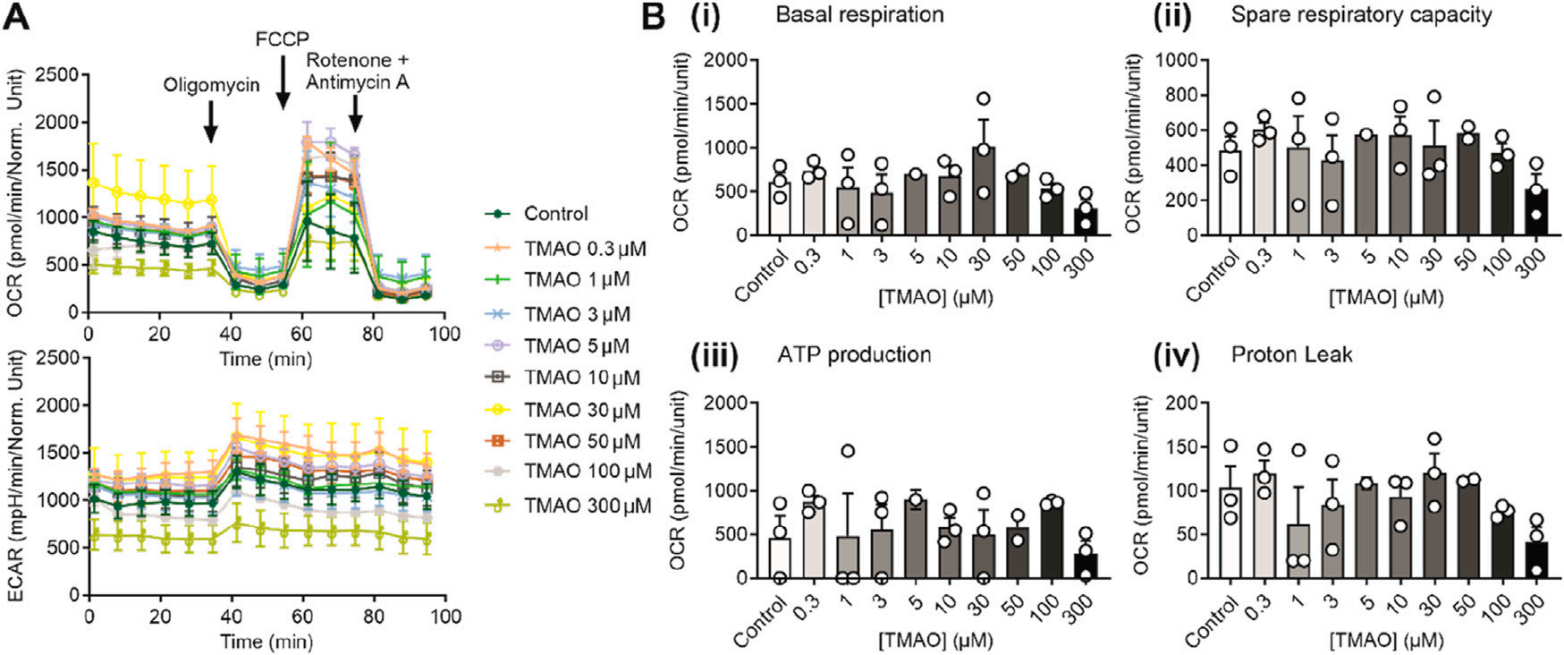
The metabolic profile of AC16 cells is unaffected by the increasing concentration of TMAO. **(A)** Real-time measurements of OCR and ECAR were measured, and oligomycin, FCCP, and antimycin A/rotenone were injected sequentially, as indicated (n = 3 experiments for each treatment). **(B)** Bar charts comparing **(i)** basal respiration, **(ii)** spare respiratory capacity, **(iii)** ATP production, and **(iv)** proton leak in AC16 cells treated with TMAO ranging from 0.3 to 300 μM (n = 3 experiments for each treatment).

Increases in TMAO caused a limitation in contraction but did not affect cell death in Figures 1–3. To assess whether this was due to a change in the metabolic profile of the cell, ATP production assays alongside seahorse respirometry, evaluation of oxygen consumption, and extracellular acidification rates were used. In these experiments in AC16 cells, there was no significant change in the oxygen consumption and extracellular acidification rates (Figure 4A), in basal respiration, spare respiratory capacity, ATP production, or proton leak (Figure 4B) with increasing TMAO concentration.

### Action potential morphology is changed by acute application of TMAO

**FIGURE 5:**
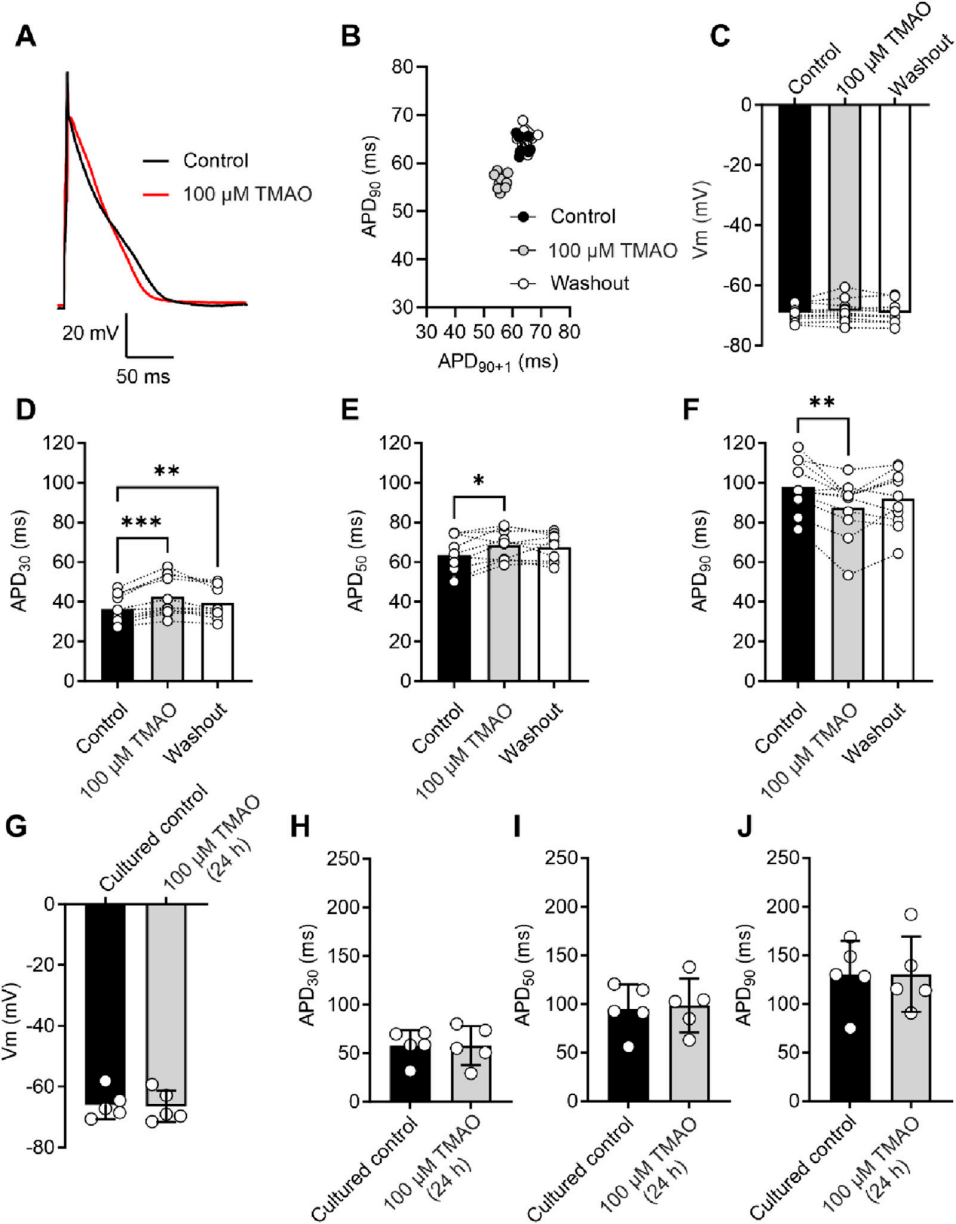
Acute treatment with 100 μM TMAO causes changes in the action potential morphology; however, 24-h treatment with TMAO has no effect on the action potential. **(A)** Example action potential recorded from a rat ventricular myocyte. Trace is the mean of 10 APs in each condition. **(B)** Poincare plot showing the AP variability of the example AP in **(A)** in control, 100 μM TMAO, and washout conditions. **(C)** Mean membrane potential in control, 100 μM TMAO, and washout showing no difference in each condition (repeated-measures ANOVA, n = 11 animals). **(F)** Mean data showing APD_30_ (**P = 0.009, ***P = 0.0002) **(D)**, APD_50_ (*P = 0.037) **(E),** and APD_90_ (*P = 0.041, **P = 0.0044) (repeated-measures ANOVA with Bonferroni’s *post hoc* test, n = 11 animals). **(G)** Mean membrane potential in cardiomyocytes cultured for 24 h in the absence (control) or presence of 100 μM TMAO, showing no significant difference (unpaired *t*-test). Mean data showing APD_30_ **(H)**, APD_50_ **(I),** and APD_90_ **(J)** in cardiomyocytes cultured for 24 h in the absence (control) or presence of 100 μM TMAO showing no significant difference (unpaired *t*-test, n = 5 (10 cells) in both group).

As increasing concentrations of TMAO had a deleterious effect on the contractile recovery in the metabolic inhibition and reperfusion protocol, it was hypothesised that higher concentrations of TMAO would alter the action potential. Given that there was a reduced contractile recovery, it was hypothesised that there would be a substantial shortening of the action potential duration (APD) to uncouple electrical activity and contraction. The action potential duration to 30% repolarised (APD_30_), APD_50_, and APD_90_ were assessed at the end of the equilibration period, following 5 min of perfusion with 100 μM TMAO, and again at the end of a 5-min washout with the NT solution (Figures 5A,B). There was no overall effect on the resting membrane potential of the cardiomyocytes (Figure 5C). There was a significant shortening of APD_90_; however, this shortening would not be expected to be sufficient to cause a loss of contractile function (Figures 5A–F). Surprisingly, there was a significant prolongation of APD_30_ (Figure 5D). These findings indicated a perturbation of the action potential, however, not enough to cause a significant loss of excitation–contraction coupling. The effects of TMAO on the APD appear to be transient. When isolated cardiomyocytes were cultured for 24 h in the absence (control) or presence of 100 μM TMAO, there was no significant effect on the membrane potential or APD_30_, APD_50_, or APD_90_ (Figures 5G–J).

### Calcium currents are potentiated by acute application of TMAO

**FIGURE 6:**
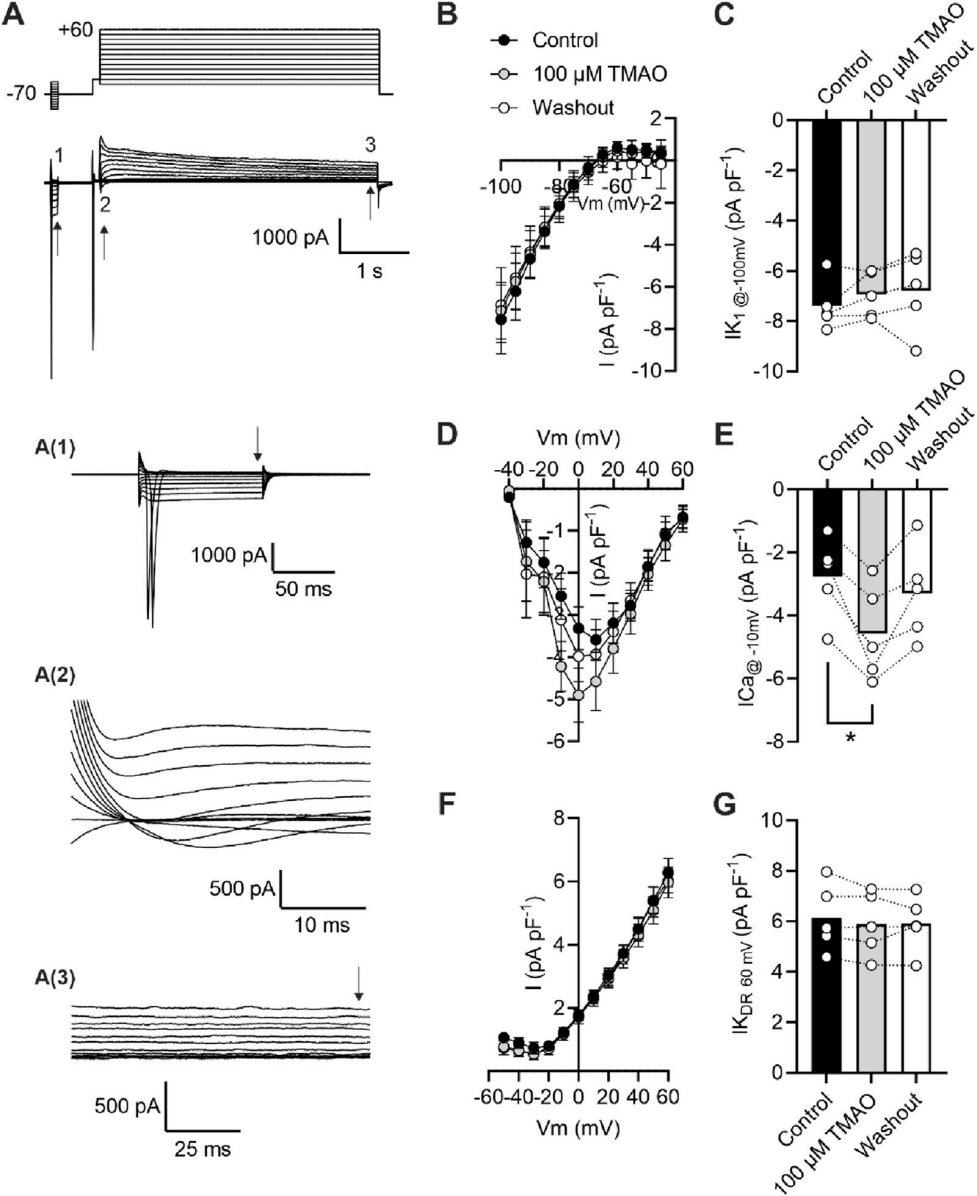
Acute treatment with 100 μM TMAO causes a significant increase in Ca^2+^ currents but no effects on inward rectifier or delayed rectifier currents. **(A)** Example trace and expanded traces of the whole cell recording protocol used to investigate **(1)** inward rectifier currents, **(2)** Ca^2+^ currents, and **(3)** delayed rectifier currents. **(B)** Mean current–voltage plot and **(C)** mean peak inwardly rectifying K^+^ current at −100 mV recorded at the point indicated with the arrow, following 5 min of equilibration in the NT solution, 5 min of perfusion with 100 μM TMAO, and after 5 min of washout with the NT solution (repeated measures ANOVA with Dunnett’s post-test, n = 5 animals (11 cells)). **(D)** Mean current–voltage plot and **(E)** mean peak inward Ca^2+^ current at −10 mV recorded in the three conditions outlined in **(C)**. *P = 0.0184, repeated measures ANOVA with Dunnett’s post-test, n = 5 animals (11 cells). **(F)** Mean current–voltage plot and **(G)** mean peak delayed rectifier K^+^ current at 60 mV recorded in the three conditions outlined in **(C)**. Repeated measures ANOVA with Dunnett’s post-test, n = 5 animals (11 cells).

**FIGURE 7:**
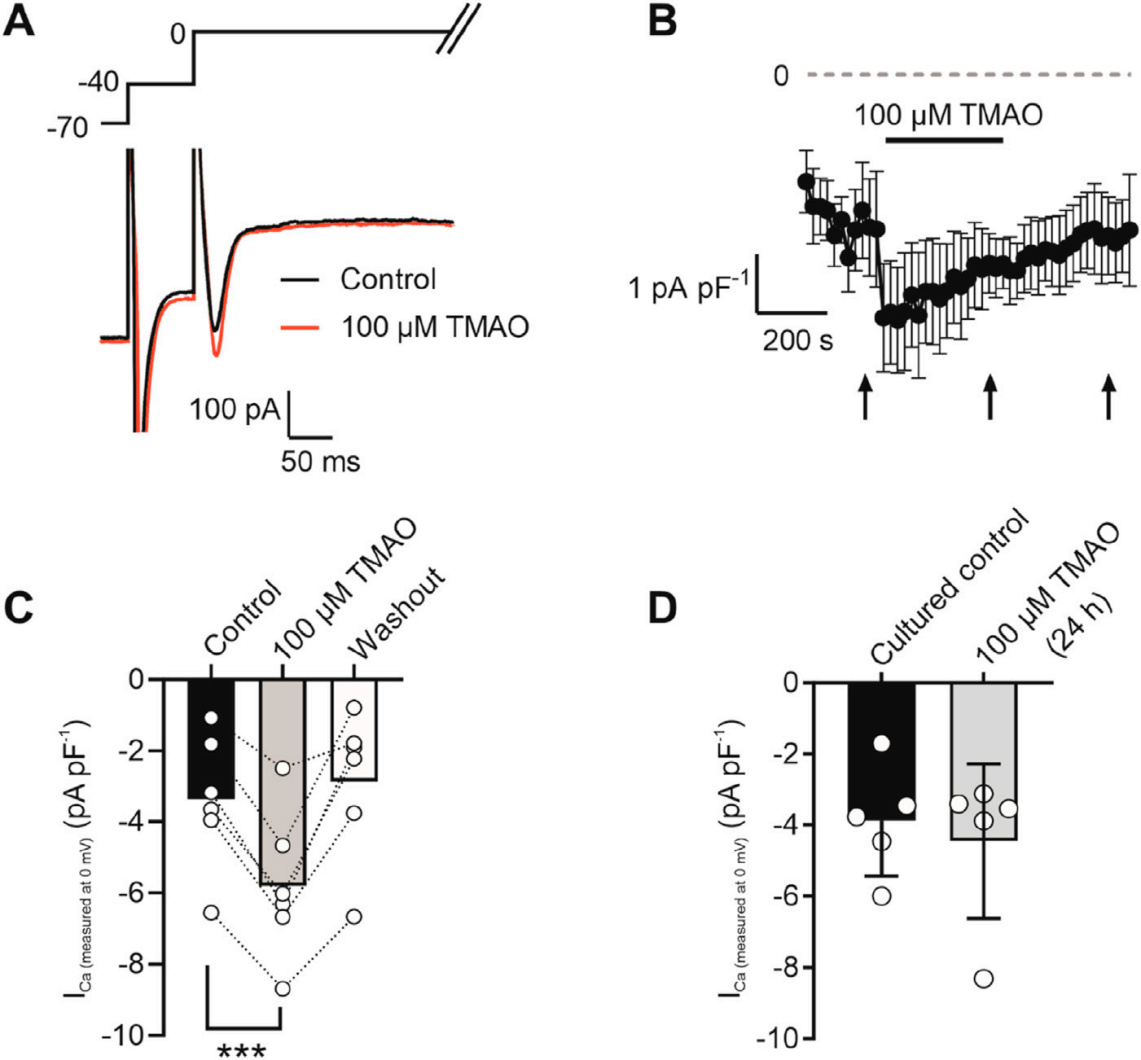
Potentiation of calcium currents by 100 μM TMAO is transient and washed out readily. **(A)** Example trace showing the voltage-step protocol and example of the current recorded in the absence (control) and presence of 100 μM TMAO. **(B)** Time course showing the mean calcium current (subtracted from the outward potassium current) throughout the recording. Arrows indicate where measurements of current were taken from the mean data displayed in **(C)**. ***P = 0.0002, Repeated measures ANOVA with Dunnett’s post-test, n = 7 animals (7 cells). **(D)** Mean Ca^2+^ current recorded in cells cultured for 24 h in the absence (control) and presence of 100 μM TMAO (unpaired *t*-test, n = 5 animals (14 and 12 cells for control and TMAO culture, respectively)).

**FIGURE 8:**
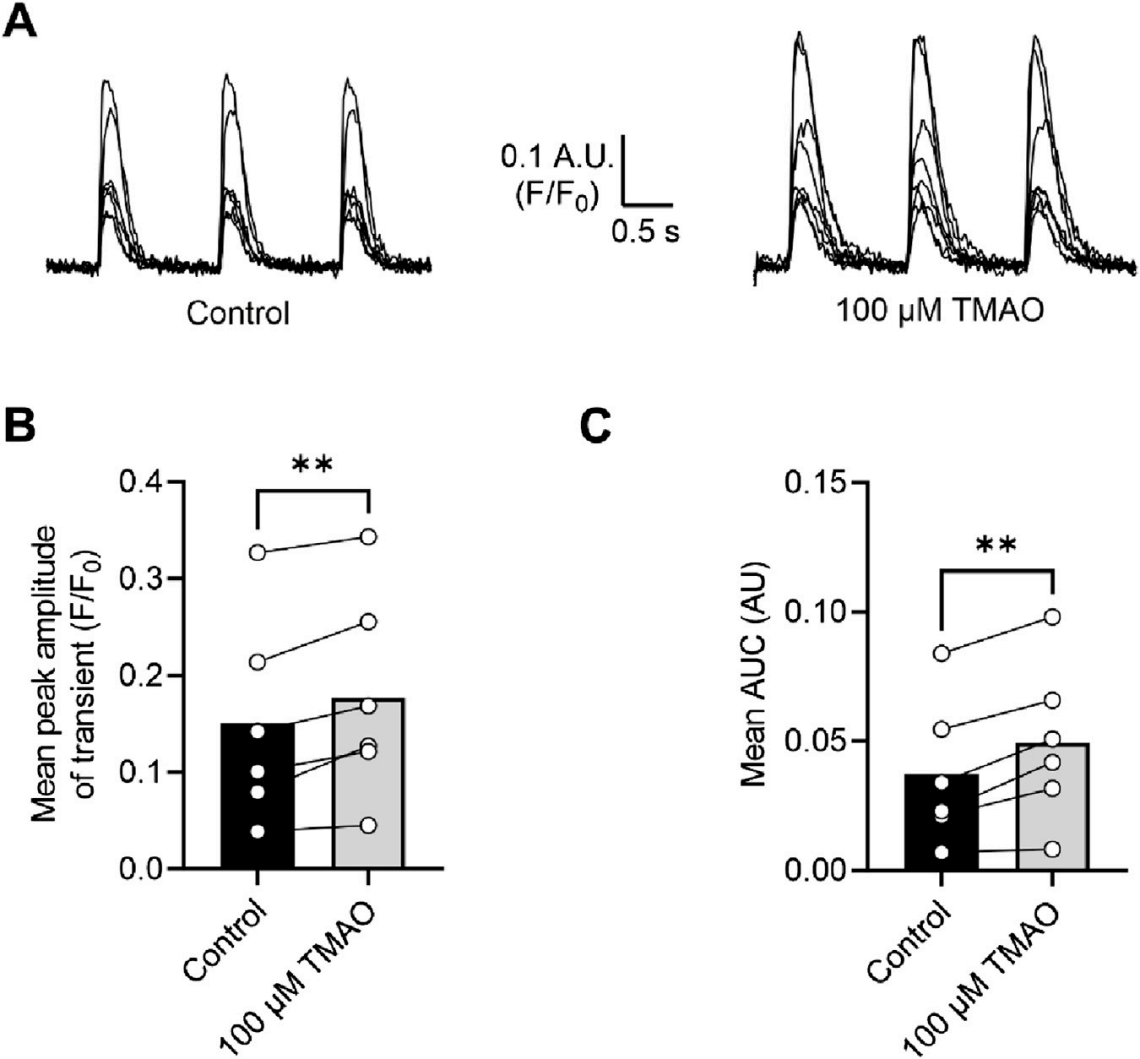
Intracellular Ca^2+^ transients are increased on treatment with 100 μM TMAO. **(A)** Example transients from eight cells in the absence and presence of 100 μM TMAO. Cells were loaded with 5 μM Fluo-4-AM, perfused at 32°C ± 2°C, and stimulated to contract at 1 Hz. The fluorophore was excited at 488 nm, and emitted light was collected above 520 nm. Example traces were taken from the end of 5 min equilibration in NT solution and following 5 min of perfusion with 100 μM TMAO. **(B)** Mean transient amplitude was increased in the presence of TMAO (**P = 0.0089, paired t-test, n = 6 animals (23 cells)), as was **(C)** the area under the curve (**P < 0.0051, paired t-test, n = 6 animals (23 cells)).

To assess changes in calcium and potassium currents with the application of TMAO, a voltage protocol was used (Figure 6A). Consistent with the membrane potential data in Figure 5C, there was no change in the mean IK_1_ current in control, TMAO, or washout conditions (Figures 6B,C). Contrastingly, there was a significant increase in the peak inward Ca^2+^ current in the presence of 100 μM TMAO, which was reversed by washout with the NT solution (Figures 6D,E). Surprisingly, given the shortening of APD_90_ observed in Figure 5F, there was not a significant increase in the delayed rectifier currents in the presence of 100 μM TMAO (Figures 6F,G). Further investigation of the change in the Ca^2+^ current over time was made using a repeating voltage-step protocol (Figure 7A). The data suggest an increased Ca^2+^ current with rapid onset with the application of 100 μM TMAO to the perfusate (Figure 7B), which remains significantly increased, following 5 min of perfusion with 100 μM TMAO, and was readily reversed, following 5 min of washout (Figure 7C). The transient nature of this increased Ca^2+^ current was further confirmed in cells that have been cultured for 24 h in the absence (control) or presence of 100 μM TMAO. There was no significant difference between the currents in treated or untreated conditions (Figure 7D). Using Ca^2+^ fluorescence measurements, the Ca^2+^-transient duration, amplitude, and area under the curve were all significantly increased in the presence of 100 μM TMAO (Figures 8A–D), consistent with the increase in the Ca^2+^ current observed in Figures 6, 7.

### Contractions in isolated cardiomyocytes are reduced by perfusion with 100 μM TMAO

**FIGURE 9:**
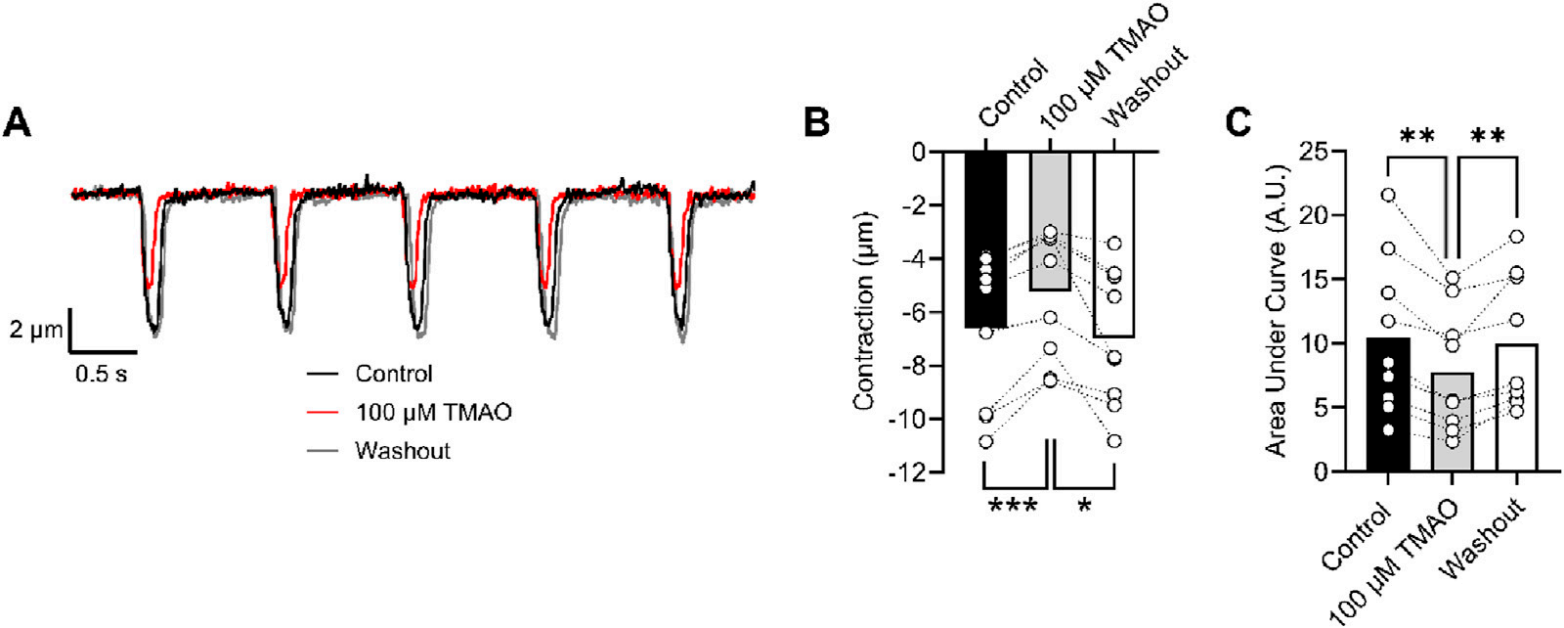
Contractile and amplitude are reduced in the presence of 100 μM TMAO. **(A)** Example traces of video edge detection recordings from a single cardiomyocyte, following 5 min of perfusion with the NT solution (control), 5 min of perfusion with 100 μM TMAO, and following 5 min of washout with the NT solution. **(B)** Contractile amplitude in μm (***P = 0.0007, *P = 0.0133, repeated measures ANOVA with Tukey’s post-test, n = 9 (39 cells)) and **(C)** area under the curve (**P = 0.004, 0.007 repeated measures ANOVA with Tukey’s post-test, n = 9 (39 cells)) were all measured.

The amplitude of cardiomyocyte contractions was measured using video-edge detection in the absence and presence of TMAO (Figure 9A). The unexpected finding was that the contractile response was both smaller in amplitude and area under the curve in the presence of TMAO (Figures 9B, C), which contrasted with the Ca^2+^ current (Figures 6, 7) and the Ca^2+^ transients (Figure 8).

### Sarcolemmal cardiac ATP-sensitive potassium (K_ATP_) channels, with a Kir6.1 pore, are potentiated by perfusion with 100 μM TMAO

**FIGURE 10:**
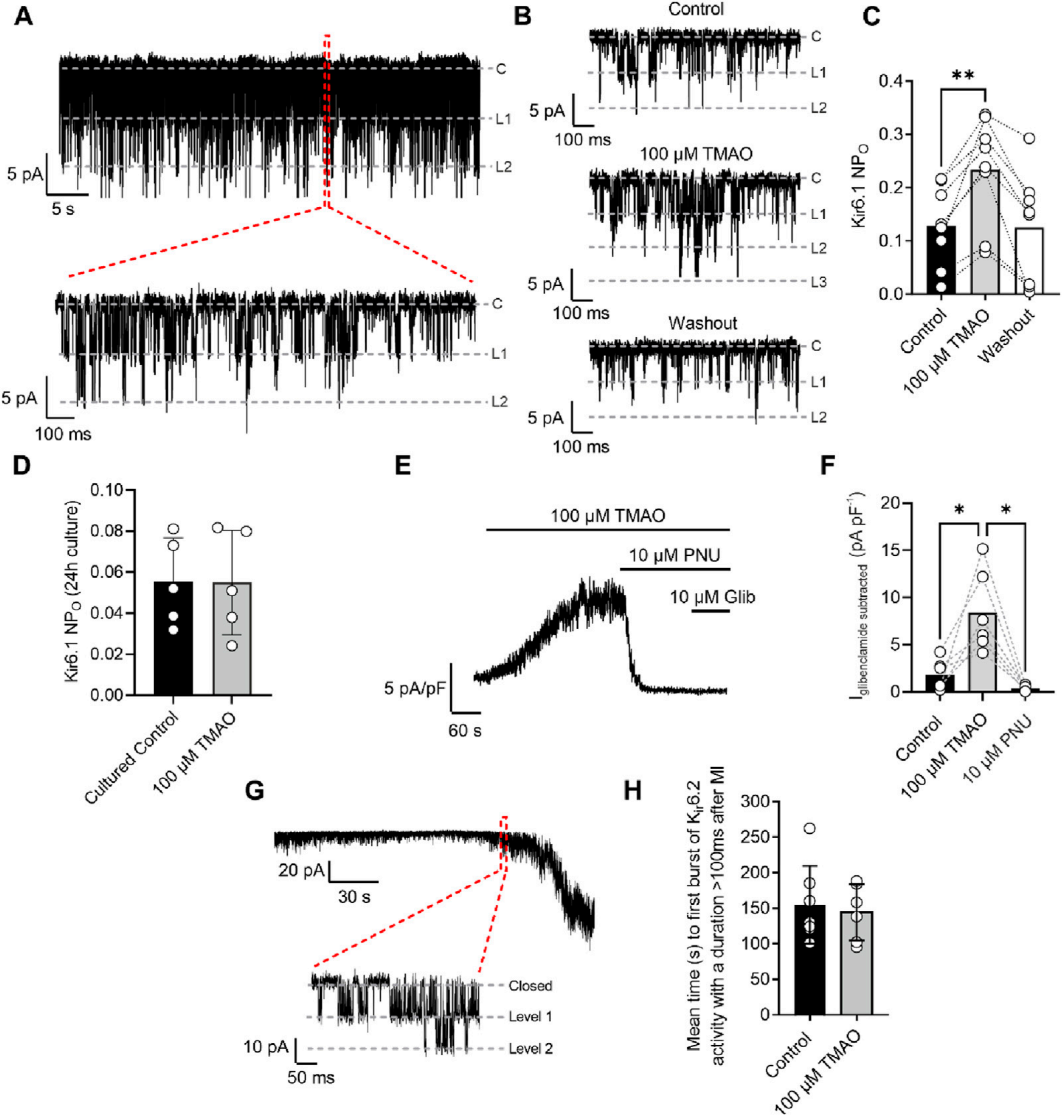
Kir6.1 channel activity is transiently increased in the presence of 100 μM TMAO. **(A)** A 30-s example trace of Kir6.1 channel activity in the cell-attached patch recording, with an expanded 1-s duration shown below, showing at least two levels of Kir6.1 activity. **(B)** Short sections of cell-attached recording of Kir6.1 channel activity in control, 100 μM TMAO, and washout, demonstrating a distinct increase in activity with 100 μM TMAO. **(C)** Mean NP_O_ of Kir6.1 activity recorded at the end of control perfusion, at the end of 100 μM TMAO and the end of washout perfusion (P = 0.0016, repeated measures ANOVA with Dunnett’s post-test, n = 8 (14 cells). **(D)** Mean Kir6.1 NPo in cardiomyocytes, following 24 h of culture in the absence (control) and presence of 100 μM TMAO (P = 0.97, unpaired t-test, n = 5 (12) and 5 (10) for control and TMAO-treated, respectively). **(E)** Example trace showing activation of human Kir6.1/SUR2B subunits stably expressed in CHO cells with 100 μM TMAO and inhibited with the Kir6.1-pore blocker PNU37883A. **(F)** Bar chart showing mean data in Kir6.1/SUR2B channels with TMAO activation and inhibition with PNU37883A. *P = 0.0247 and 0.0167 for control vs TMAO and TMAO vs PNU respectively, repeated Measures ANOVA with Dunnett’s post-test, n = 6 passages (11 cells). **(G)** Cell-attached patch recording in the presence of SFT-MI solution with inset showing the activation of the metabolically sensitive Kir6.2 channel. **(H)** Bar chart showing the mean time to Kir6.2 opening in the SFT-MI inhibition solution in the absence (control) and presence of 100 μM TMAO. Meanwhile, there was no significant difference in Kir6.2 activation (P = 0.70, unpaired t-test, n = 7 (12 cells) and 6 (12 cells) for control and TMAO respectively).

The cardiac sarcolemmal K_ATP_ channel has always been described as a highly ATP-sensitive current that opens in response to catastrophic ATP depletion to preserve remaining ATP (Brennan et al., 2024; Brennan et al., 2015). Recently, a second cardiac K_ATP_ channel complex has been identified at the surface of cardiomyocytes, consisting of a Kir6.1 pore-forming subunit that displays constitutive, rather than ATP-dependent, activity (Brennan et al., 2024). Kir6.1 constitutive activity was seen throughout the recording (Figure 10A: 30-s example section of recording) but was increased at the end of 5 min of perfusion with 100 μM TMAO (Figures 10B,C). The increased NPo with TMAO was reversed, following 5 min of washout with the NT solution. To assess the effects of chronic treatment with 100 μM TMAO on Kir6.1 constitutive activity, cardiomyocytes were cultured for 24 h in the absence (control) or presence of TMAO. Cell-attached recording of both groups showed no significant difference in the mean NPo between the two groups, once more suggesting that TMAO has a transient effect on cardiomyocytes (Figure 10D).

To further investigate whether TMAO can activate Kir6.1 channels, a CHO cell line stably expressing Kir6.1/SUR2B was used. This approach was chosen due to the complexity of isolating Kir6.1 whole-cell currents in cardiomyocytes and the challenges in pharmacologically distinguishing between Kir6.1 and Kir6.2 currents. On application of 100 µM TMAO, a robust whole-cell current was observed. This current was inhibited fully by the Kir6.1-selective pore blocker PNU37883A with no additional inhibition in the presence of glibenclamide, a pan-K_ATP_ channel family blocker (Figures 10E,F) (Brennan et al., 2024).

To determine whether there were any effects of TMAO on the canonical Kir6.2/SUR2A complex, metabolic inhibition was applied to cardiomyocytes in the absence (control) or presence of 100 μM TMAO, and the time taken to activate this current was measured. The time to current activation was unaffected by 100 μM TMAO (Figures 10G,H), suggesting little modulation of this channel complex or, similar to data in Figure 4, alterations to ATP synthesis, given this channel’s role as an ATP sensor (Brennan et al., 2024; Brennan et al., 2015).

**FIGURE 11:**
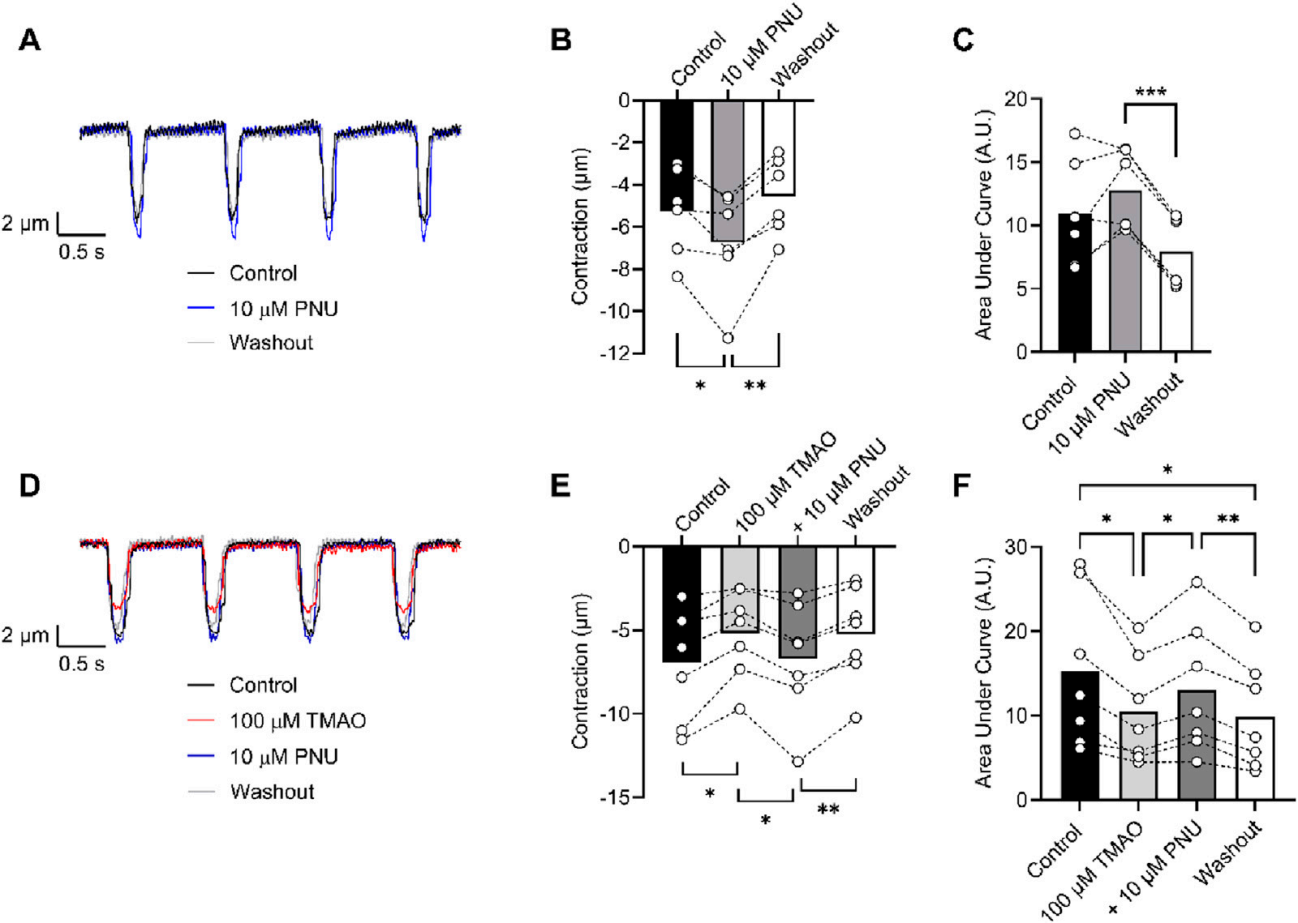
Contractile function reductions by TMAO can be reversed by selective inhibition of Kir6.1 ion channels with PNU37883A. **(A)** Example traces showing the effect of the selective block of Kir6.1 using 10 μM PNU37883A, showing an increase in contractile amplitude. Mean **(B)** contractile amplitude (*P = 0.041, **P = 0.008, repeated measures ANOVA with Tukey’s post-test, n = 6 animals) and **(C)** area under the curve (***P < 0.0001, repeated measures ANOVA with Tukey’s post-test, n = 6 animals) showing an increase in contractile amplitude in the presence of PNU37883A. **(D)** Example traces showing the effect of 100 μM TMAO and TMAO with 10 μM PNU37883A. **(E)** Mean data showing contractile amplitude in the presence of 100 μM TMAO, 100 μM TMAO with 10 μM PNU37883A, and washout (*P = 0.018 and 0.017, **P = 0.005 repeated measures ANOVA with Tukey’s post-test, n = 7 animals). **(F)** Mean data showing the area under the curve in the conditions outlined in D and E (*P = 0.031 (Control vs. TMAO), *P = 0.021 (TMAO vs. PNU), **P = 0.005 (PNU vs. Washout), and *P = 0.036 (Control vs. Washout), repeated measures ANOVA with Tukey’s post-test, n = 7 animals).

To determine whether activation of Kir6.1 in cardiomyocytes was responsible for the reduction in contractile efficacy identified in Figure 9, experiments were carried out in the presence of 10 µM PNU37883A to inhibit the Kir6.1 current in ventricular myocytes in the presence of 100 μM TMAO. In the absence of TMAO, PNU37883A was able to increase the contractile amplitude, as previously reported (BRENNAN ET AL 2024). In the presence of 100 μM TMAO, PNU37883A was able to attenuate the reduction in the contractile function in cardiomyocytes (Figure 11).

## Discussion

The cardiovascular effects of TMAO have long been debated. This study found no adverse impact of TMAO on mitochondrial energy production or the infarct size. Rather, TMAO altered contractile function by modulating the action potential, enhancing both the L-type Ca^2+^ current and the Kir6.1 current in ventricular myocytes, even at the highest concentrations. Elevated plasma TMAO levels are linked to major adverse CVD events (Tang and Hazen, 2014; Schuett et al., 2017; Lau et al., 2017), and this link has been studied in different cohorts (Schuett et al., 2017; Troseid et al., 2015). Although TMAO may be both a marker and mediator of lifestyle diseases, some studies suggest it also play a protective role, indicating that the correlation between elevated TMAO levels and CVD risk is complex (Nowinski and Ufnal, 2018). Therefore, a better understanding of TMAO’s biological role in health and disease is crucial to determine its feasibility as a therapeutic target or routine biomarker.

In this study, we found that TMAO had limited effects on cardiomyocyte survival and viability; however, the potentiation of the L-type Ca^2+^ and Kir6.1 currents was clearly seen in the action potential. This manifested as a prolongation of APD_30_ with a shortening of APD_90_ (Figure 5). Although such a modulation of the action potential might be deemed to have minimal effects on the contractile function of cardiac cells, it is evident that TMAO is transiently limiting the amplitude of contraction as the washout of TMAO reverses the dampening of contractions (Figure 8). It is, perhaps, somewhat surprising that a small shortening of the action potential would cause such a profound effect on the contractile response in cardiomyocytes, but the kinetics of the Ki6.1 channel complex should be considered in this scenario. Our group recently identified the Kir6.1 channel in cardiomyocytes (Brennan et al., 2024). This channel is weakly rectifying, meaning it remains open across the voltage range of the cardiac action potential. In contrast, the strongly rectifying Kir2. x channels, which underlies the cardiac IK_1_ current, are nearly completely blocked by intracellular polyamines and Mg^2+^ at membrane potentials more positive than approximately −20 mV. It is plausible that the opening of Kir6.1 channels across the voltage range could inhibit the cardiac action potential under normal physiological conditions (Figures 5, 8, 9). This may also explain the loss of contractile function during metabolic inhibition (Figures 1, 2), where the Kir6.2/SUR2A channels, the well-established canonical cardiac K_ATP_ channel subunits, would also be open (Brennan et al., 2024; Brennan et al., 2015; Baier et al., 1994). The loss of contractile function is interesting, given that calcium cycling has increased in amplitude; however, the inhibitory effect of the increased K^+^ current on contraction cannot be underestimated.

Our findings suggest a limited effect of TMAO on mitochondrial function, or on ATP levels in the cell, exemplified by our recordings, showing no change to the time of Kir6.2/SUR2A activation. Our findings are in disagreement with some findings in the literature, where TMAO was suggested to disrupt cardiac mitochondrial oxidative phosphorylation (Makrecka-Kuka et al., 2017). It should be noted that in our study, there was an acute change in TMAO to reflect the cycling of TMAO concentration within the blood, whereas in other studies, more long-term treatment of TMAO has been used. Furthermore, our respirometry experiments were carried out in AC10 cells. To obtain enough cells to give accurate respirometry experiments, a monolayer is the most accurate. In our case, acutely isolated cardiomyocytes are dissociated and a heterologous population that contains 70%–90% rod-shaped healthy cardiomyocytes, however, will also contain some dead cells, cell debris, and other cell types, such as fibroblasts. Here, this resulted in variable respirometry data t and is something that we would like to improve in the future. Our Kir6.2/SUR2A data support the findings in the AC10 cells, where it was no different in the time to activation of K_ATP_ channels in metabolic inhibition.

Unlike the Kir6.2/SUR2A channel complex, which is fully inhibited until substantial ATP depletion, Kir6.1 channels open with a constitutive activity, suggesting a limited ATP sensitivity (Brennan et al., 2024). The increased NPo value of the Kir6.1 channel in the presence of TMAO suggests that there is a pathway that leads to Kir6.1 potentiation, either by direct modulation or by cell signalling. The activation of the Kir6.1 current was seen using cell-attached patch recording with 100 μM TMAO (Figure 10). The Kir6.1 channel openings were characterized by their amplitude (∼5 pA at −110 mV) and distinct bursting characteristics, unlike the more prolonged openings of the Kir2. x channels underlying IK_1_. In whole-cell recordings in CHO cells stably expressing human Kir6.1/SUR2B, a similar potentiation of the current was seen with 100 μM TMAO, suggesting that TMAO either interacts directly with the channel or through an unidentified modulator activated by TMAO (Figure 10).

Evidence for an “uncoupling” of contractile function, rather than the death of cardiomyocytes, comes from the cell survival data, which shows that although the TMAO concentration is increased, there was no reduction in cell survival despite a loss of contractile recovery (Figure 1). Contractile recovery following metabolic inhibition and washout, as used in Figures 1, 2, is widely used, by us and others, as part of the assessment of the cardioprotective or cardiotoxic properties of compounds toward myocytes, alongside measurements of cell survival (Brennan et al., 2019; Brennan et al., 2015; Rodrigo et al., 2004; Rodrigo and Standen, 2005; Turrell et al., 2011). In previous applications of this protocol, decreased cell survival is coupled with a decreased contractile recovery, indicating cardiotoxicity. However, in contrast here, loss of contractile recovery appears to be uncoupled from cell survival (Figure 1). There was no evidence for a worsened outcome (reduced cell survival, Figure 1), worsened infarct size (Figure 3), or in the assessment of mitochondrial energy production with metabolic challenges (Figure 4). Despite this, there was a marked reduction in contractile function, which would normally be categorised as a cardiotoxic effect.

The potentiation of the L-Type Ca^2+^ channel seen with 100 μM TMAO (Figures 6, 7) would be expected to lead to an increase in cytoplasmic Ca^2+^ accumulation during each contractile cycle. In these experiments, there was an increase in the amplitude of the amplitude and area under the curve of the Ca^2+^ transients, consistent with an increased Ca^2+^ current, leading to an increased Ca^2+^-induced Ca^2+^ release (Figure 8). This increased [Ca^2+^]_i_ change during each transient was coupled with a *reduction* in the contractile parameters, a somewhat surprising finding. Activation of the Kir6.1 complex with pharmacological modulation by pinacidil in cardiac cells is known to cause a reduction in contractile function, as demonstrated in our previous publications; however, it is normally coupled with a *reduced* Ca^2+^ transient amplitude, duration, and AUC (Brennan et al., 2024; Brennan et al., 2015). Our findings are in common with other reports in the literature, where the increase in TMAO concentration leads to a significant prolongation of the rate of relaxation, following a contraction, and a reduction in the maximal rate of shortening and the amplitude of shortening (Savi et al., 2018).

To assess whether selective inhibition of the Kir6.1 channel did improve the contractile function in the presence of 100 μM TMAO, cells were perfused with PNU37883A, a Kir6.1 pore-blocking compound (Figure 11). In these experiments, in the absence of TMAO, PNU37883A was able to enhance the contractile function of cardiomyocytes, agreeing with our previous finding of constitutive activity of Kir6.1 currents that is part of the normal physiological modulation of cardiac function. In the presence of 100 µM TMAO, PNU37883A was able to reverse the loss of contractile function, consistent with a TMAO concentration-dependent role for inducing Kir6.1 channel potentiation in dampening the contractile function in cardiomyocytes.

The data presented in this study suggest that TMAO may not be *acutely* damaging to cardiac tissue in terms of increasing propensity to ischaemia but may have deleterious effects on contractile function. We found no evidence that mitochondrial function was impaired in the presence of 100 μM TMAO. Despite this, there were changes to contractile function that could be, at least in part, attributed to the potentiation of Ca^2+^ and Kir6.1 currents. Importantly, these effects did not seem to occur with chronic treatment with TMAO, only acutely. It is plausible that the cycling of TMAO, following meals, could have a detrimental effect on cardiac function in patients with excessively high TMAO concentrations in blood; however, our findings suggest that these patients would not be more likely to suffer from increased myocardial damage in the short term. This disruption of normal myocardial contractile function is perhaps more concerning. Given that the chronic effects of TMAO on channel potentiation are absent, it would be important for future studies to investigate the cycling of TMAO concentrations, following food intake to investigate potential changes in myocardial function with acute elevations of blood TMAO levels.

### Limitations

A potential limitation to our experiments in cellular models is the slightly lower than physiological temperature used in electrophysiology, contractile function, and calcium imaging experiments. At temperatures above 34°C, bubbles can form in the tubing of our perfusion systems. These bubbles can disrupt recordings by knocking cells as they exit into the bath chamber, causing artefacts, loss of seal in patch recording, or complete loss of cells from the field of view in imaging and edge-detection experiments. To avoid these issues, we maintained the temperature below 34°C, which is a necessary compromise applied to all our cellular experiments.

It should be noted that this is an *in vitro*/*ex vivo* study, and therefore, some of the confounding influences of TMAO may be missing in these *ex vivo* models, such as the influence of TMAO on the vasculature, endothelial responses, and the immune system. It is increasingly clear that TMAO has deleterious effects on the liver and also on endothelial cells. Therefore, future work in this area should further investigate the heart–liver axis in terms of the hepato- and cardiotoxicity of TMA and TMAO. Furthermore, the effects of the TMAO on the cardiac and vascular endothelial layer are absent in this study as most work has been carried out in isolated cardiomyocytes. To fully assess the effects of TMAO on membrane currents, it has been necessary to carry out experiments in an *in vitro* setting, as is a common compromise of research models characterising electrophysiological modulation of currents.

A further limitation to the study is that the work only included male-derived cardiomyocytes. Our group has limited the use of female-derived cells in work that investigates cardioprotection because female pre-menopausal hearts have shown significant intrinsic protection (Bendale et al., 2013; Jovanovic et al., 2000; Ma et al., 2009; Murphy et al., 2011). In this study, data investigating the non-cardioprotected cells and comparing them to cells protected by ischaemic preconditioning are not possible in females as we observed that ischaemic preconditioning does not further increase the cardioprotective phenotype, specifically the time to contractile failure and increased contractile recovery and cell survival in our metabolic inhibition and washout data. In future studies, it will be important for us to characterise the effects in both male- and female-derived tissue.

## Conclusions

These data lead us to conclude that TMAO may not be causing poor cardiovascular health but may be a potential biomarker for suboptimal dietary and lifestyle choices, leading to poor gut microbiota, which could suggest an elevated risk of cardiovascular disease. Our findings cannot rule out that the poor function seen in our cellular models could lead to worsened outcomes for patients in terms of functional recovery after acute myocardial infarction, an increased propensity for arrhythmias, and further detrimental reduction in function in patients with heart failure syndrome.

## Data Availability

The raw data supporting the conclusions of this article will be made available by the authors, without undue reservation.

## Abbreviations

APD, action potential duration; APD_90_, action potential to 90% repolarisation; K_ATP_ channel, ATP sensitive potassium channel; Po, channel open probability; NPo, channel open probability with more than one level of opening; TMA, trimethylamine; TMAO, trimethylamine N-oxide.
